# Modulation of miR-21 signaling by MPS1 in human glioblastoma

**DOI:** 10.18632/oncotarget.4143

**Published:** 2015-05-15

**Authors:** Uday B. Maachani, Anita Tandle, Uma Shankavaram, Tamalee Kramp, Kevin A. Camphausen

**Affiliations:** ^1^ Radiation Oncology Branch, National Cancer Institute, National Institutes of Health, Bethesda, Maryland, USA

**Keywords:** glioblastoma multiforme, MPS1, MiR21, PDCD4, MSH2, TGF-β/SMAD signaling

## Abstract

Monopolar spindle 1 (MPS1) is an essential spindle assembly checkpoint (SAC) kinase involved in determining spindle integrity. Beyond its mitotic functions, it has been implicated in several other signaling pathways. Our earlier studies have elaborated on role of MPS1 in glioblastoma (GBM) radiosensitization. In this study using reverse phase protein arrays (RPPAs), we assessed MPS1 mediated cell signaling pathways and demonstrated that inhibiting MPS1 could upregulate the expression of the tumor suppressor PDCD4 and MSH2 genes, by down regulating micro RNA-21 (miR-21). In GBMs miR-21 expression is significantly elevated and is associated with chemo and radioresistance. Both MPS1 and miR-21 depletion suppressed GBM cell proliferation, whereas, ectopic expression of miR-21 rescued GBM cell growth from MPS1 inhibition. Further, we demonstrate that MPS1 mediates phosphorylation of SMAD3 but not SMAD2 in GBM cells; A possible mechanism behind miR-21 modulation by MPS1. Collectively, our results shed light onto an important role of MPS1 in TGF-β/SMAD signaling via miR-21 regulation. We also, show the prognostic effect of miR-21, PDCD4 and MSH2 levels to patient survival across different GBM molecular subtypes. This scenario in which miR-21 is modulated by MPS1 inhibition may be exploited as a potential target for effective GBM therapy.

## INTRODUCTION

Glioblastoma multiforme or glioblastoma (GBM) continues to be the most frequently diagnosed and lethal primary brain tumor. Patients have a median survival of less than 15 months following standard of care [1]. Low survival rates are attributable to the aggressiveness of GBM and a lack of understanding of the molecular mechanisms underlying its progression. Deregulation of kinase-mediated signal transduction is implied in GBM tumorigenesis [2, 3]. Previously, we performed a siRNA-based RNAi screen focused on the human kinome to identify protein kinases required for the survival of GBM [4] and identified Monopolar spindle 1 (MPS1 also known as TTK) as a putative target for GBM therapy and demonstrated MPS1 inhibition radiosensitizes GBM cells by abrogating DNA repair and as a consequence, cells eventually undergoing mitotic catastrophe [5]. MPS1 mitotic kinase is an evolutionary conserved protein kinase that is overexpressed in several human cancers and most widely functions in cell cycle control, including mitotic spindle assembly checkpoint activation, proper mitotic progression, centrosome duplication, chromosome alignment, error correction of kinetochore-microtubule attachment, and recruitment of SAC components to kinetochores [6, 7]. It is located predominantly in the cytoplasm during interphase and relocates to the nucleus late in G2 phase and then associates with the kinetochore from prophase to metaphase [8, 9].

Beyond mitosis MPS1 kinase has been implicated, in genotoxic stress response, such as stress caused by DNA damage [10, 11], in development, cytokinesis, and several different signaling pathways [12], like non-canonical Smad signaling pathway, wherein activation of Mps1 promotes Transforming Growth Factor-β-independent Smad signaling [13]. Genetic and pharmaceutical blockades of Mps1 kinase are known to induce tumor cell death while leaving the untransformed normal cell unaffected [7, 14] making it an ideal candidate for GBM therapy. Recent results from at least one MPS1 inhibitor, NMSP715, showed great promise in preclinical cancer models [5, 15].

In the present study using Reverse phase protein arrays (RPPAs) and bioinformatics approach, we assessed the MPS1 mediated cell signaling pathways in GBM. Our results demonstrate that MPS1 inhibition results in induction of tumor suppressor PDCD4 and MSH2 expression through modulation of oncogenic miR-21 via a non-canonical Smad signaling pathway.

## RESULTS

### Proteomic profiling in MPS1 inhibited GBM cells using reverse phase protein arrays (RPPA) and Ingenuity Pathway Analysis (IPA)

**Figure 1:**
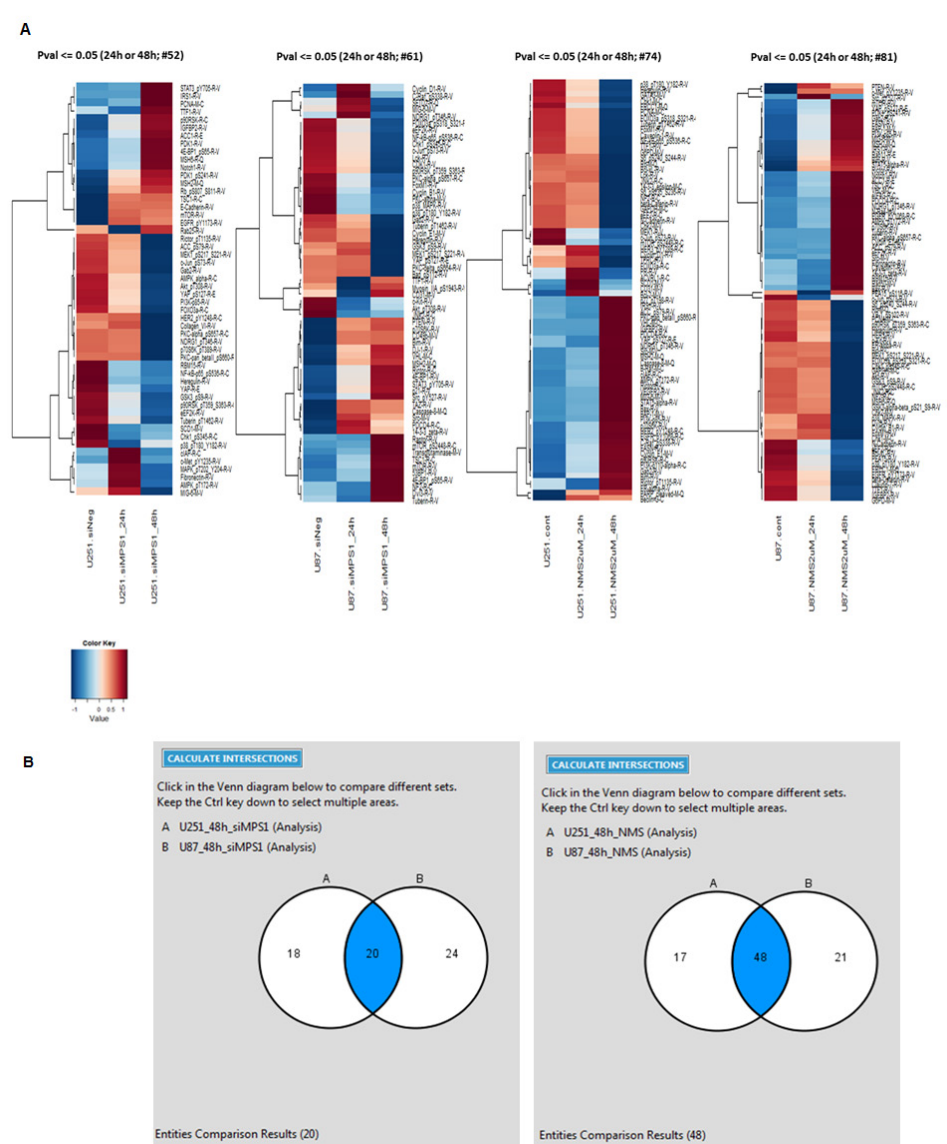
Proteomic profiling in MPS1 inhibited GBM cells using reverse phase protein arrays (RPPA) Panel **A.** represent protein intensity values log2 and z-score transformed as fold change FC > 1.2 (Red) FC < 1.2 (Blue) with reference to siMPS1 and NMSP715 treated U251, U87 cells at 24hrs and 48hrs **B.** Venn diagrams presenting common molecules effected under MPS1 inhibition between U251 and U87cells either treated with siMPS1 or NMSP715 at 48hr time point.

To determine the biological effects of MPS1 inhibition on signaling pathways in GBM, we profiled the modulation of phosphorylated and non-phosphorylated proteins using RPPA. We compared the levels of 172 phosphorylated and non-phosphorylated proteins in MPS1 inhibited (RNAi or NMSP715 (an ATP-competitive inhibitor of MPS1 recently developed and characterized [5, 15]) U251, U87 GBM cells. Analyses revealed significantly (*P* < 0.05) differentially expressed proteins between normal and MPS1 inhibited cells (Figure 1A). Comparison analysis using Ingenuity Pathway Analysis (IPA) software was performed to analyze the biological states between U251, U87 cells in both RNAi and Drug (NMSP715) mediated inhibition of MPS1. We identified 20 proteins in siMPS1 and 48 proteins in drug treated U251 and U87 cells commonly affected (Figure 1B). The cellular signaling pathways for each group of genes under MPS1 inhibition, and the top canonical pathways found included: *PI3/AKT signaling, Neurogulin signaling, ErbB signaling, GBM signaling, UVB induced MAPK signaling, mTOR signaling and Molecular mechanism of cancer* (Table 1). Analysis predicted miR-21-5p (miR-21), REL, TGFB1, EGR1, AGT, PTEN as top regulator effect networks and their downstream targets with in the dataset (Table 1). Regulator Effects analytic in IPA provide insight into the causes and effects of differentially expressed genes or proteins in a dataset and explains how predicted activated or inhibited upstream regulators might cause increases or decreases in phenotypic or functional outcomes downstream. Further a predicted molecular interaction network of these commonly affected proteins with miR-21 was created. We identified two tumor suppressor genes PDCD4 and MSH2 within the dataset associated with miR-21 and elevated under MPS1 (siMPS1 and NMP715 mediated) inhibition (Sup Figure 1A, 1B). Taken together, these results suggest a probable role of MPS1 in regulation of tumor suppressor PDCD4, MSH2, which are direct targets of oncogenic miR-21.

**Table 1:** Ingenuity Pathway Analysis (IPA): Top Canonical Pathways and Top regulator effect networks. Represent Ingenuity Pathway Analysis (IPA) of cellular signaling pathways for each group of genes under MPS1 inhibition, predicting significant top canonical pathways and their top regulator effect networks (represent predicted activated or inhibited upstream regulators based on their downstream targets)

**Summary of Analysis - U251_48h_siMPS1** **Top Canonical Pathways** **Name** **p-value** PI3K/AKT Signaling 3.32E-14 ErbB Signaling 6.32E-14 mTOR Signaling 6.88E-14 Neuregulin Signaling 8.73E-14 UVB-Induced MAPK Signaling 9.04E-14	**Top Regulator Effect Networks** **Regulators Consistency Score** REL 0.894 TGFB1 -10.205
**Summary of Analysis - U87_48h_siMPS1** PI3K/AKT Signaling 7.19E-19 Neuregulin Signaling 6.8E-17 UVB-Induced MAPK Signaling 3.05E-15 Molecular Mechanisms of Cancer 1.81E-14 Glioblastoma Multiforme Signaling 2.29E-14	miR-21-5p 1.500
**Summary of Analysis - U251_48h_NMS**PI3K/AKT Signaling 8.5E-20 Neuregulin Signaling 3.18E-20 ErbB Signaling 1.35E-18 Molecular Mechanisms of Cancer 9.96E-26 Glioblastoma Multiforme Signaling 1.35E-18	None Regulators Found
**Summary of Analysis - U87_48h_NMS** PI3K/AKT Signaling 4.68E-16 Neuregulin Signaling 7.93E-20 p70S6K Signaling 3.37E-16 Molecular Mechanisms of Cancer 7.53E-18 Glioblastoma Multiforme Signaling 1.4E-16	EGR1 2.000 AGT 2.000 PTEN 1.414

### MPS1 inhibition induces PDCD4 and MSH2 expression through modulating miR-21 levels

**Figure 2:**
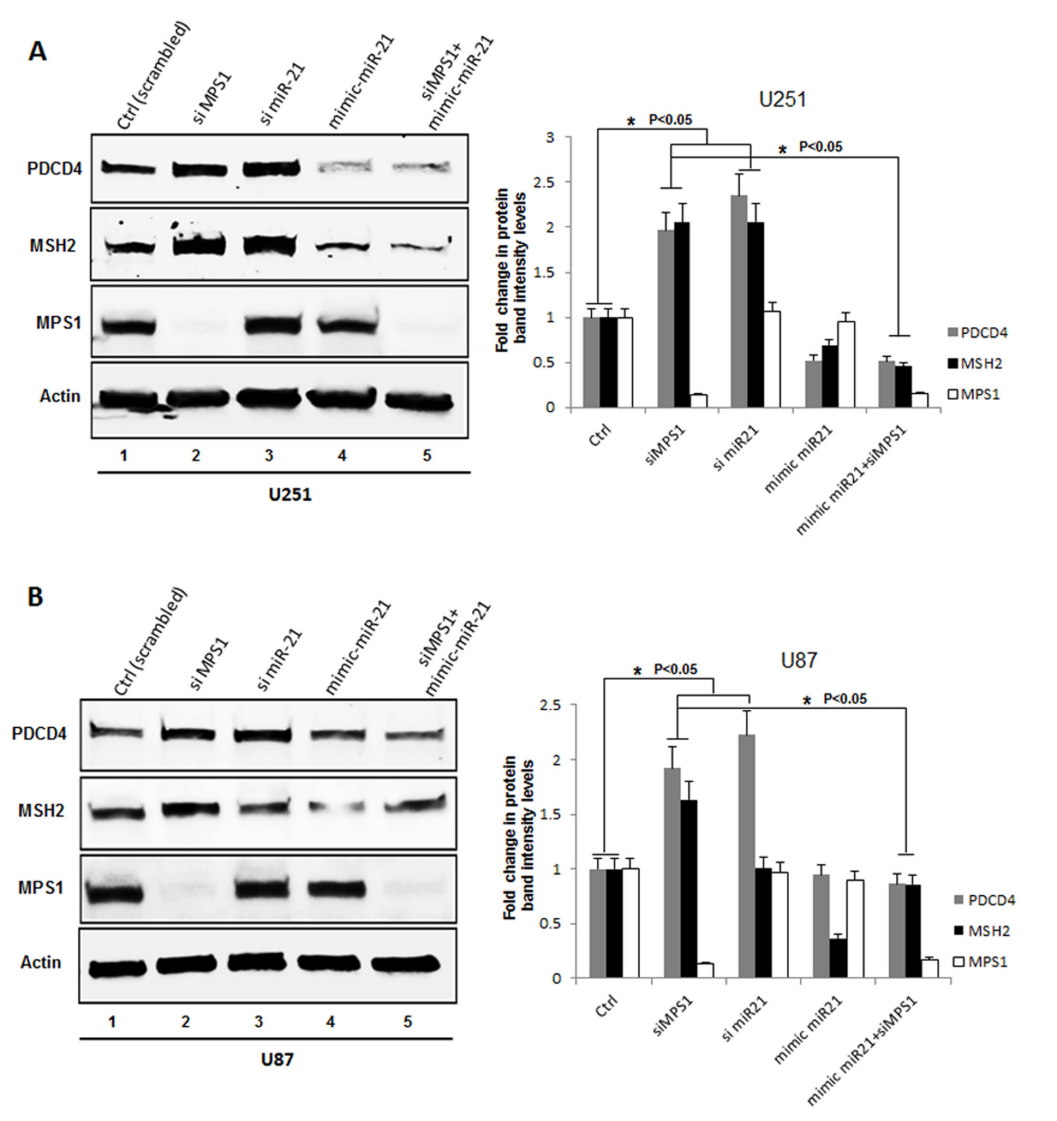
Genomic silencing of MPS1 enhances PDCD4 and MSH2 expression *in vitro* **A.**, **B.** Western blot analysis of PDCD4, MSH2, MPS1 and β-Actin proteins from cell lysates of U251 and U87 treated GBM cells as indicated at 48hr time point, with their corresponding bar graphs of western blots representing the fold change in protein band intensities normalized to β-Actin quantified densitometrically using Image-J software NIH. Data presented are the mean ± S.D. Student's *t* test was performed and the level of significance * indicate *p* < 0.05.

**Figure 3:**
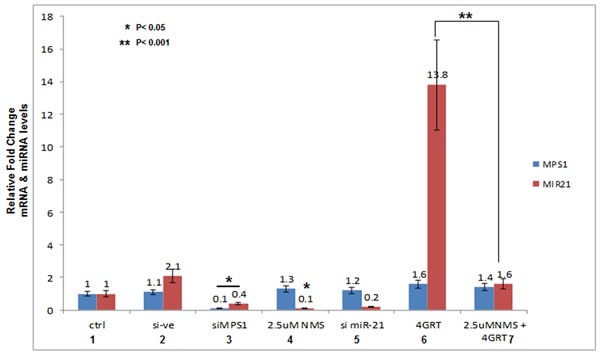
MPS1 modulates miR-21 expression Total RNA from U251 GBM cells was extracted at 48hrs after indicated treatments and cDNA was synthesized. Figure 3 represent RT- PCR values as fold change (2^^CT-method ) of miR-21-miRNA transcript levels normalized to U6snRNA and MPS1 transcript levels normalized to GAPDH. Data presented are the mean ± S.D. Student's t test was performed and the level of significance *indicate *p* < 0.05, **p < 0.001.

Next we confirmed modulation of tumor suppressor PDCD4, MSH2 levels on MPS1 inhibition by immunoblot analysis. GBM cells U251, U87 were either transfected with siMPS1 or treated with NMSP715. Protein lysates were prepared after 48hrs treatment, immunobloted and probed for PDCD4, MSH2 and MPS1. We see significant increase in PDCD4 and MSH2 protein levels under siMPS1 (*P* < 0.05) (lane 2 Figure 2A, 2B) and NMSP715 (lane 2 Sup Figure 2A, 2B) treated U251, U87 GBM cells. Since PDCD4 and MSH2 are direct targets of miR-21 (which targets the 3’ untranslated region of their mRNAs and represses their expression), we analyzed their levels under RNAi mediated miR-21 inhibition along with its ectopic expression by miR-21 mimic (hmiR-21-5p) transfection. The results clearly show a significant increase in PDCD4 (*P* < 0.05 both in U251 & U87 cells) and MSH2 (*P* < 0.05 in U251 cells alone) levels after miR-21 knockdown (lane 3 Figure 2A, 2B) and a decrease with miR-21 mimic transfection (lane 4 Figure 2A, 2B). While ectopic expression of miR-21 (mimic) in MPS1 knockdown cells significantly (*P* < 0.05) repressed induction of PDCD4 and MSH2 (lane 5 Figure 2A, 2B). These results clearly indicate both MPS1 and miR-21 modulate PDCD4 and MSH2 expression. To study if these two events are linked, we performed RT-PCR to quantify miR-21 levels under MPS1 inhibition in U251 cells. MPS1 inhibition significantly (RNAi (*p* < 0.05) (lane 3, Figure 3) or NMSP715 (*p* < 0.05) (lane4, Figure 3)) depleted miR-21 levels. However si miR-21 did not affect MPS1 expression either at protein (lane 3 Figure 2A, 2B) or at transcript levels (lane 5, Figure 3) indicating miR-21 is downstream of MPS1. Many lines of evidence suggest up regulation of miR-21 in response to ionizing radiation (IR) and its role in chemo and radio resistance of tumor cells [15, 16]. In view of these reports, we sought to find if MPS1 inhibition abrogates radiation induced miR-21 expression. Not surprisingly, NMSP715 significantly (*P* < 0.001) repressed IR induced miR-21 levels in U251 GBM cells (lane7, Figure 3). Together, these results provide evidence, suggesting that MPS1 is upstream of miR-21, and regulates PDCD4 and MSH2 via miR-21 modulation.

### MPS1 and miR-21 inhibition affects GBM cell proliferation; while ectopic-expression of miR-21 protect GBM cells from MPS1 inhibition

**Figure 4:**
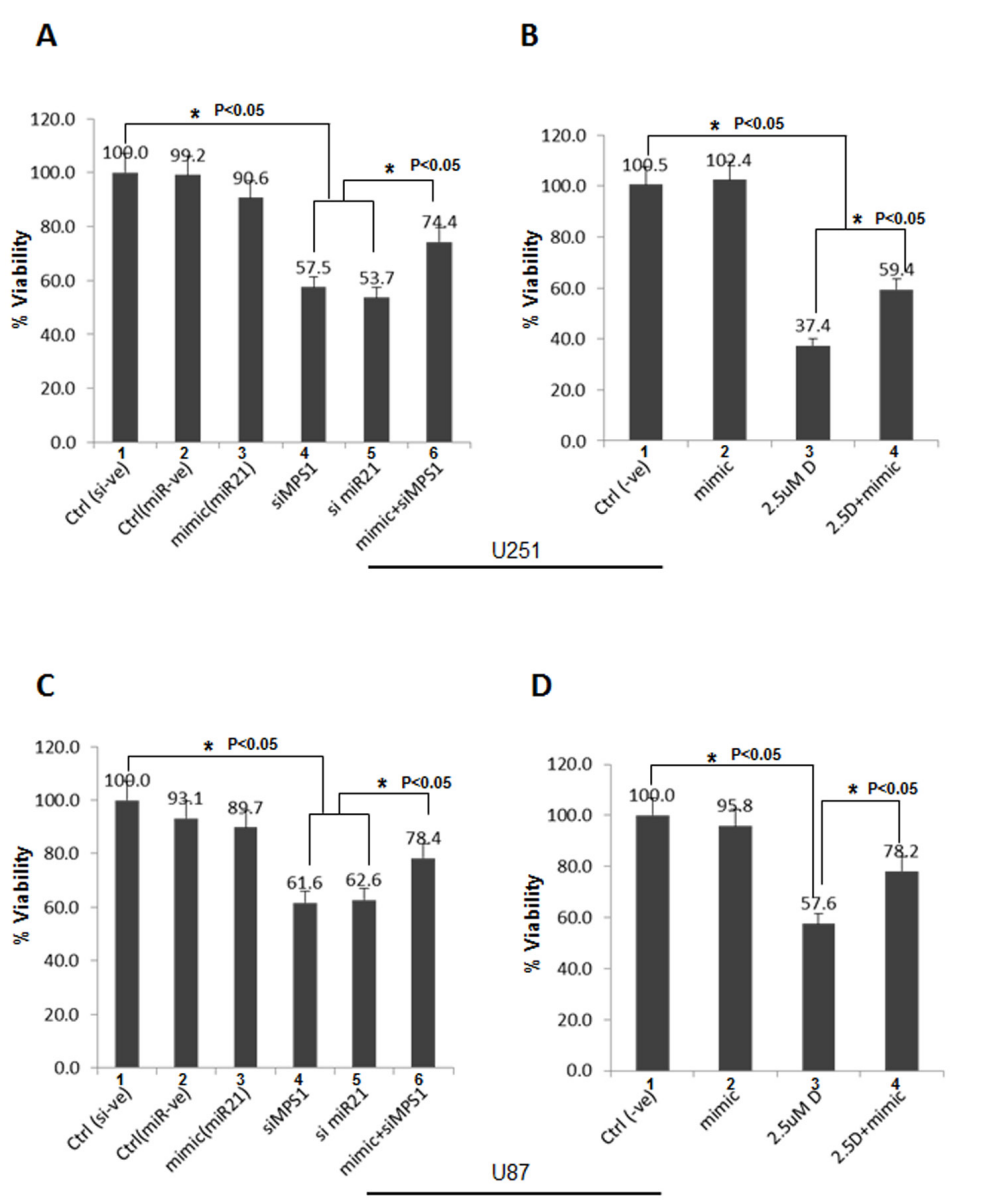
Ectopic miR-21 expression protects GBM cells from MPS1 inhibition induced cell death The human GBM cell lines U251 and U87 were transfected with si miR-21, siMPS1 and miR-21 mimic alone or co-transfected together as indicated in triplicates. For NMSP715 treated cells miR-21 mimics were transfected 2 hours prior drug treatment. Cell viability (Cell Titer Glo) was assessed after 5 days post treatments. scrambled siRNA and micro RNA mimic –ve controls were used. **A., B.** Represent bar graph % viability for U251 cells. **C.**, **D.** Bar graph % viability for U87 cells. Data presented are the mean ± the standard deviation relative to control transfected cells. Student's t test was performed and the level of significance * indicate *p* < 0.05.

Previously we have reported genetic and pharmaceutical blockades of MPS1 inhibit GBM cell proliferation by induction of mitotic catastrophe [5] and miR-21 has been shown to act as an anti-apoptotic factor in glioblastoma-derived cell lines [18 ,19]. Since, as demonstrated earlier, the endogenous miR-21 levels are depleted after MPS1 inhibition. Here, we hypothesized that its ectopic expression might confer protective effect on GBM cell survival. Analysis of survival rates by Luminescent cell viability assay showed a significant (*P* < 0.05) decrease in GBM cell proliferation (both U251 and U87) with MPS1 (siMPS1 (lane 4, Figure 4A, 4C) or NMSP715 (lane 3, Figure 4B, 4D)) or miR-21 (si miR-21) inhibition (lane 5, Figure 4A, 4C). Consistent with our hypothesis ectopic expression of miR-21 mimics after MPS1 inhibition (siMPS1 (lane 6, Figure 4A, 4C) or NMSP715 (lane 4, Figure 4B, 4D)) significantly (*P* < 0.05) but not totally rescued GBM cell attenuation. Notably, ectopic expression of miR-21 mimic alone had no negative effect on GBM cell growth (lane 3, Figure 4A, 4C & lane 2, Figure 4B, 4D), consistent with earlier reports [20]. These results clearly demonstrate MPS1 role in modulating miR-21 and GBM cell survival.

### MPS1 inhibition with NMSP715 induces tumor suppressor PDCD4 , MSH2 in GBM tumors *in vivo*

**Figure 5:**
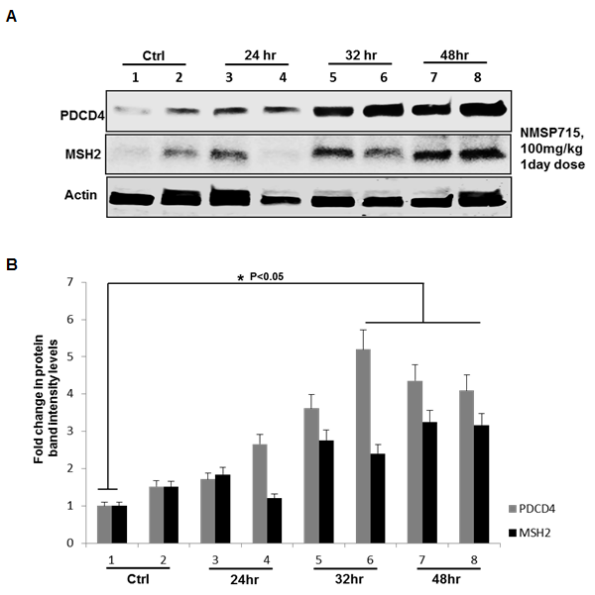
Pharmacologic MPS1 inhibition induces tumor suppressor PDCD4, MSH2 in GBM tumors *in vivo* U251 subcutaneous tumors were treated with NMSP715 (NMS) (100mg/kg). One day after treatment tumors were extracted at indicated timepoints and tumor lysates were subjected to western blots. Panel **A.** represent western blots of U251 tumor lysates, (lanes 1-2) untreated tumors, (lanes 3-8) NMSP715 treated tumors from different mice. **B.** corresponding bargraph representing the fold change in protein band intensities normalized to β-Actin quantified densitometrically using Image-J software NIH. Data presented are the mean ± S.D. Student's *t* test was performed and the level of significance * indicate *p* < 0.05.

Previously we have reported MPS1 abrogation inhibits GBM tumor growth *in vivo, with* an enhanced radiation-induced tumor growth delay [5]. We here, next determined if the in *vitro* results would mimic in *in vivo*. Mice bearing U251 GBM tumors (~140 mm3) were randomized into two groups, vehicle treated controls and NMSP715 100 mg/kg (was delivered oral gavage). One day after drug treatment the mice were sacked and tumors were isolated at different time points. We noticed significant (*P* < 0.05) increase in PDCD4 and MSH2 levels at later time points with NMSP715 treatment (Figure 5A, 5B). These findings, further confirm MPS1 role in miR-21 modulation and its targets in an *in vivo* setup.

### MPS1 inhibition effects phophorylation of SMAD3; a possible mechanism behind miR-21 regulation

**Figure 6:**
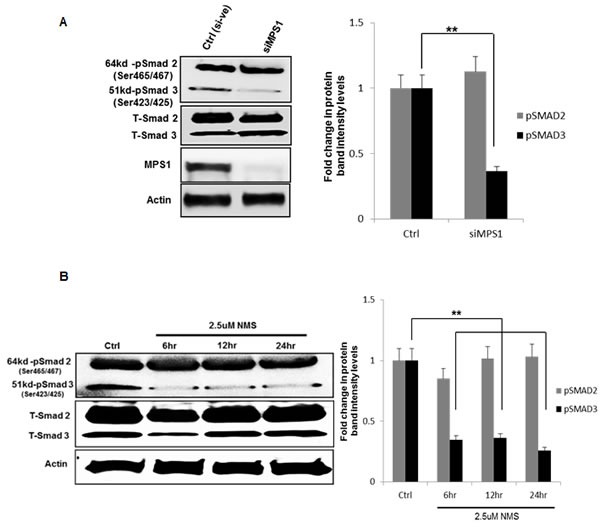
MPS1 inhibition affects phophorylation of SMAD3 not SMAD2 U251 GBM cells were treated either with siRNA or NMSP715 and cell lysates were immune probed for total and phopho SMAD2/3. **A.** represent westernblots of siMPS1 treated U251 cells at 48hr timepoint, **B.** western blots of NMSP715 treated U251cells at 6h, 12hr, and 24hr time points along with corresponding bargraphs representing fold change in protein band intensities of phospho-SMAD2 and SMAD3 (pSMAD2/3) normalized to β-Actin, quantified densitometrically using Image-J software NIH. Data presented are the mean ± S.D. Student's *t* test was performed and the level of significance ** indicate *p* < 0.005

**Figure 7:**
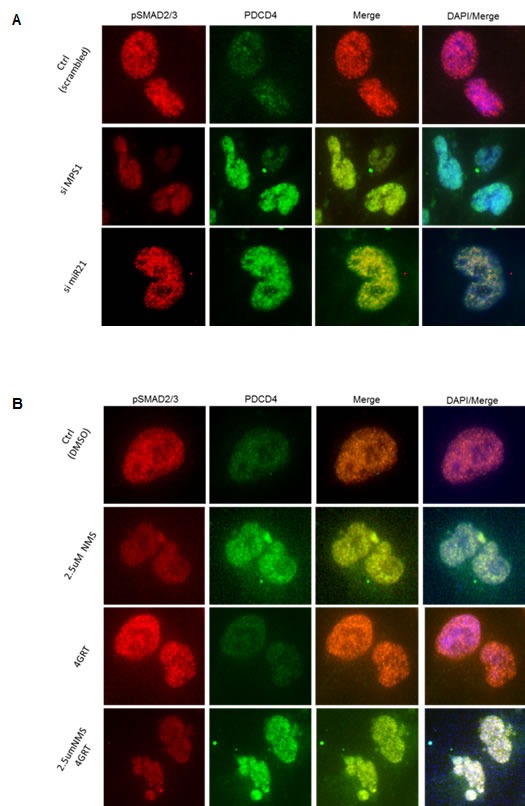
MPS1 inhibition affects nuclear localization of phopo SMAD2/3 Panel **A., B.** represent the immunofluorescence images of treated (as indicated) U251 cells at 48hr time point probed for pSMAD2/3 (red), PDCD4 (green), DAPI (blue). The results clearly demonstrate siMPS1 or NMSP715 inhibits SMAD3 phosphorylation and its subsequent localization into nucleus.

To characterize the molecular mechanism underlying MPS1 role in MiR-21 modulation, we focused on the demonstrated miR-21 role in targeting tumor suppressor PDCD4 and MSH2 [21, 22] in various cancer and GBM cells [17]. Earlier studies have reported TGF-β/Smad signaling regulates miR-21 expression [23, 24], and Smad3, but not Smad2 signaling mediates increased expression of miR-21 [25]. More recently MPS1 has been implicated in TGF-β independent activation of SMAD signaling, via phosphorylation of Smad2 and Smad3 (but not Smad4) at the SSXS motif in their C-terminal regions *in vitro* and *in vivo* [26, 27]. Phosphorylation of Smad2 and Smad3 (SMAD2/3) enables them to partner with Smad4 and translocate to the nucleus (as a complex) [28], where they regulate transcription of target genes. Based on these reports, here we examined phosphorylation status of SMAD2, SMAD3 in MPS1 inhibited U251 cells. The results in (Figure 6A, 6B) clearly show significant (*P* < 0.005) decrease in phospho (p) SMAD3 (but not pSMAD2) both under RNAi and NMSP715 mediated inhibition of MPS1. Immunofluorescence studies also show an increase in PDCD4 levels and decrease in pSMAD2/3 levels in the nucleus of MPS1 inhibited U251 cells (Figure 7A, 7B). While, miR-21 knockdown showed substantial increase in PDCD4 levels, but did not affect nuclear localization of pSMAD2/3 (Figure 7A). Together, these findings provide evidence that MPS1 modulates miR-21 via SMAD3 phosphorylation. More studies are required to delineate the exact role of MPS1 in selective phosphorylation of SMAD3 and its effect on TGF*-β/SMAD* signaling pathway.

### High expression of miR-21 and low expression of PDCD4, MSH2 effects overall survival in GBM patients

We next evaluated the prognostic effect of miR-21, PDCD4 and MSH2 expression on survival in GBM patient samples from TCGA database (https://tcga-data.nci.nih.gov). Using in house Glioblastoma Bio Discovery Portal (GBM-BioDP-(http://gbm-biodp.nci.nih.gov)) [29], multivariate cox proportional hazards model survival analysis was carried out examining miR-21, PDCD4 and MSH2 expression (red- above median expression, blue- below median expression) and overall survival association in different subclasses (C-classical, M-mesenchymal, P- proneural and N- neural) of GBM (197 patients), along with their expression profile as Box plots (Figure S4). The results clearly indicate high expression profile of miR-21 across different GBM subtypes, with poor prognosis for survival with high expression, however, only the Proneural subtype reached the statistical significance (logrank p-value 0.008), where it showed the widest range of miR-21 expression (Figure S4A). PDCD4 showed low expression profile and followed better prognosis for survival trend with high expression in Mesenchymal (significant logrank p-value 0.001), Proneural and Neural GBM subtypes (Sup Figure 4B, 4C). While high expression of MSH2 showed significantly better prognosis for survival in Classical (logrank p-value 0.012) and Mesenchymal (logrank p-value 0.003). We also constructed expression correlation heatmaps between miRNAs and gene products (PDCD4, MSH2) among GBM patients (*n* = 197), which clearly show miR-21 anti-correlated to PDCD4, MSH2 expression (Sup Figure 4D). These results clearly imply an inverse correlation between miR-21 verses PDCD4 and MSH2 expression and point them out as suitable bio-markers for GBM disease progression.

## DISCUSSION

Previously in our lab and others have demonstrated MPS1 as a potential target for cancer therapy [30-32] and we successfully demonstrated MPS1 enhances radiosensitivity of human GBM cells by modulating DNA repair [5]. In the present study using Reverse phase protein arrays (RPPAs) [33] and bioinformatics approach, we assessed the biological effects of MPS1 inhibition on signaling pathways. Monopolar spindle 1 (MPS1) is an essential spindle assembly checkpoint (SAC) kinase having functions beyond its multiple roles in mitosis [6-9]. It has been implicated in development, cytokinesis, genotoxic stress response and several different signaling pathways [10-12]. In this study we show that MPS1inhibition induces enhanced expression of tumor suppressor PDCD4 and MSH2 genes (Figure 1 & Figure 2). Programmed cell death 4 (PDCD4) is a newly identified tumour suppressor and has been demonstrated to inhibit neoplastic transformation [34] and its loss of expression is associated with glioblastoma [35]. MutS homolog 2 (MSH2) is a main member of the DNA mismatch repair (MMR) system, which is essential for genome stability and recombination of chromosomes [36]. Decreased expression of MSH2 is associated with promoting carcinogenesis by accelerating the accumulation of mutations in oncogenes and tumor suppressor genes [37]. Over expression of these two genes (PDCD4, MSH2) has been shown to cause tumor suppression by inducing apoptosis in cancer cells [38, 39]. Both PDCD4 and MSH2 are direct targets of microRNA-21 (miR-21) (binds to 3’ untranslated regions (UTRs) of their mRNA's), where it post-transcriptionally down regulates their expression [22, 40, 41]. miR-21 is one of the most studied miRNAs in cancer, described as oncogenic microRNA (Oncomir), implicated in various aspects of carcinogenesis, including cellular proliferation, apoptosis, and migration [42]. Substantial data indicate that miR-21 is significantly elevated in glioblastoma (GBM) and in many other tumors of various origins [43-46]. Knockdown of miR-21 in cultured glioblastoma cells triggers activation of caspases and leads to increased apoptotic cell death [47]. miR-21 has been shown to be elevated with ionizing radiation and mediate radiation resistance of glioblastoma cells by regulating PDCD4 and MSH2 [17]. Not surprisingly in our studies, the enhanced levels of PDCD4 and MSH2 were associated with decreased miR-21 levels (Figure 2 & Figure 3), similar to earlier reports. This observation led us to hypothesize a possible link between MPS1 and miR-21 modulation. Consistent with the prediction, we observed depletion of miR-21 levels with MPS1 inhibition. Notably, miR-21 knockdown had no effect on MPS1 expression (Figure 3), suggesting miR-21 is downstream of MPS1. To explore the relevance of MPS1 and miR-21 to GBM cell survival, we performed functional rescue experiment by ectopically expressing miR-21 in MPS1 inhibited GBM cells. Ectopic expression of miR-21 significantly (*p* < 0.05) rescued anti proliferative effect of MPS1 inhibition on GBM cells (Figure 4), further confirming the upstream modulatory effect of MPS1 on miR-21 expression. We further verified these findings *in vivo* in U251 xenografts, which showed similar enhanced expression of PDCD4 and MSH2 after MPS1 inhibition with NMSP715 treatment (Figure 4). These results put together clearly demonstrate that MPS1 inhibition induces tumor suppressor PDCD4 and MSH2 through miR-21 modulation. Inhibiting miR-21 has been shown to have a potential for broad, anti-tumor effects by targeting multiple signaling pathways [48]. Our data are the first to demonstrate the role of MPS1 in miR-21 modulation, and the availability of small molecule inhibitors for MPS1 makes it an ideal therapeutic target for GBMs and other cancers.

To further characterize the molecular mechanism underlying MPS1 role in miR-21 modulation, we focused on the demonstrated miR-21 role in targeting tumor suppressor PDCD4 and MSH2 [21, 22]. Recent advances in the study of TGF-β biology have shown that TGF-β/Smad signaling regulates several microRNAs (miRNAs) including miR-21 [23, 24, 49]. Smad3, but not Smad2 has been shown to increase expression of miR-21 [25]. More recently MPS1 has also been implicated in TGF-β independent activation of SMAD signaling, via phosphorylation of Smad2 and Smad3 (but not Smad4) at the SSXS motif in their C-terminal regions *in vitro* and *in vivo* [26, 27]. Based on these reports, It is reasonable to hypothesize a possible role of MPS1 mediated phosphorylation of SMAD 2/3 and miR-21 regulation. Consistent with the above reports we observed significant (*p* < 0.05) decrease in phospho (p) SMAD3 levels (but not pSMAD2) with MPS1 inhibition in GBM cells (Figure 6). Phosphorylation of Smad2 and Smad3 enables them to partner with Smad4 and translocate to the nucleus (as a complex) [28], where they regulate transcription of target genes (Illustrative image Sup Figure 3). Evidently, we observed decrease in nuclear pSMAD2/3 levels with MPS1 inhibition, but not under miR-21 depletion (Figure 7A) consistent with earlier reports (miR-21 is downstream of SMAD signaling) [25]. Even though SMAD2 and SMAD3 are reported to be substrates for MPS1 [26, 27], we did not notice significant inhibition of phosphorylation of SMAD2 in GBM cells after MPS1 inhibition (Figure 6), contrary to earlier reports. This discrepancy can be attributed to the differential regulation and non-overlapping roles of SMAD2 and SMAD3 [50, 51] and a possible role of MPS1 in their selective phosphorylation. Since therapeutic targeting of the TGF-β/SMAD signalling pathway are being pursued , revealing the identity of factors that modulate the relative activation of Smad2 or Smad3 may provide target(s) for more effective strategies for cancer therapy. Many questions remain regarding Smads’ activities, inside and outside of canonical TGF-β signaling. More studies are needed how MPS1 can modulate selective activation of Smad2/3, which are largely unknown. We circumvented from further discussing, as it is not the scope of this study.

Earlier we reported low MPS1 expression to be a significant marker of better prognosis in GBM, breast and lung cancer [5]. To understand the prognostic clinical value of miR-21, PDCD4 and MSH2 expression in GBM patient survival, we performed Multivariate survival analysis on 197 GBM patients, using a in house Glioblastoma Bio Discovery Portal (GBM-BioDP) [29]. To date, studies have shown high miR-21 expression is correlated with high GBM pathological grades, with overall patient survival for those with low miR-21 expression to be significantly longer than those patients with high miR-21 expression [52]. PDCD4 as a diagnostic for human cancer staging and prognostic for survival in colon, lung, liver, breast, glioma and esophageal cancers has been reported earlier [38,53-57]. Consistent with earlier findings, we similarly report high expression of miR-21 in different GBM subtypes ((C)Classical (M)esenchymal , (P)roneural and (N)eural as per verhaak et.,al [58] associated with poor prognosis for patient survival (Figure S4A). While tumor suppressors PDCD4, MSH2 showed low expression across different GBM subtypes. High expressing PDCD4, MSH2 GBM patients showed better survival compared to Low expressing patients (Sup Figure 4B, 4C) and had an inverse correlation to miR-21 expression (Sup Figure 4D). Thus, miR-21, PDCD4 and MSH2 expression may serve as potential biomarkers for overall survival prediction and prognosis in GBM patients. Any clinical therapeutic intervention that might repress oncogenic miR-21 or induce tumor suppressors PDCD4 and MSH2 would certainly be a suitable candidate for GBM therapy. As mentioned, we demonstrated MPS1inhibition induces tumor suppressor PDCD4 and MSH2 through modulating oncogenic miR-21 expression. Further our results verify the multiple roles of MPS1 beyond mitosis, shedding light onto an important role of it in TGF-β/SMAD signaling. More studies are needed to understand the exact role of MPS1 in modulating other possible signaling pathways and cancer progression. It is evidently a potential target not only for effective GBM therapy but other cancers as well.

## MATERIALS AND METHODS

### Cell lines & drugs

U251, U87 (National Cancer Institute Frederick Tumor Repository) human GBM cell lines were grown in Dulbecco's Modified Eagle Medium (DMEM) (Invitrogen, Carlsbad, CA) with 10% fetal bovine serum (FBS), and maintained at 37°C, 5% CO2. NMSP715 was obtained from Calbiochem., U.S.A (cat: 475949). Drug was reconstituted in dimethyl sulfoxide (DMSO) and stored at -20 C.

### RNAi transfections and cell viability

As described earlier (5) siRNA transfections, 2-pmol siMPS1, (5’ TTGGACTGTTATACTCTTGAA3’, SI00071624, si miR-21 (GeneSolution siRNA cat: 1027416 (mix of 4 validated anti Hs_miR-21)) (Qiagen Inc., Germantown, MD) was complexed with RNAi Max lipid transfection reagent (Invitrogen) in DMEM media for 15 minutes at ambient temperature. Two thousand cells suspended in DMEM supplemented with 20% FBS were then added. (For NMSP715 mediated inhibition of MPS1, cells were plated Overnight prior to drug treatment and treated with NMSP715 at the concentrations indicated in each experiment). Plates were maintained at ambient temperature for 15 minutes before being placed at 37 C/5% CO2. Cell viability was assessed five days post siRNA transfection through quantification of ATP (CellTiter-Glo luminescent Reagent, Promega, Madison, WI). Untransfected cells and wells transfected with negative (All-star siNegative [siNeg], Qiagen) and positive (All star siCelldeath, Qiagen) control siRNAs were used as controls. Proteins for Western blot analysis was harvested 48 hours post siRNA transfection or Drug treatments.

### Transfection of MiR-21(has-miR-21-5p) mimic

U251, U87 GBM cells were transfected as above with 3 μl Lipofectamine RNAiMAX (Life Technologies; Carlsbad, CA) mixed with 30 nM meridian mimic hsa-miR-21-5p (cat: C-301023-01-0002: UAGCUUAUCAGACUGAUGUUGA) or negative control mimic (cat: CN-002000-01-05) (Dharmacon, ThermoFisherScientific; Pittsburgh PA). hsa-miR-21-5p targets PDCD4 and MSH2 (TarBase) [59].

### RPPA analysis and bioinformatics

Treated GBM cell (U251,U87) lysates were prepared in RPPA lysis buffer [1% Triton X-100, 50 nmol/L Hepes (pH 7.4), 150 nmol/L NaCl, 1.5 nmol/L MgCl2, 1 mmol/L EGTA, 100 nmol/L NaF, 10 nmol/L NaPPi, 10% glycerol, 1 nmol/L phenylmethylsulfonyl fluoride, 1 nmol/L Na3VO4, and aprotinin 10 μg/mL) as described elsewhere (37), and sent to RPPA Core Facility, MD Andersen Cancer Center, Houston, TX for RPPA analysis. Briefly, 5 serial dilutions of lysates were arrayed on nitrocellulose-coated slides, probed with (172 phosphorylated and non- phosphorylated) antibodies, and visualized by DAB colorimetric reaction [29]. Relative protein levels for each sample were determined by interpolation of each dilution curves from the standard curve antibody slide. All the data points were normalized for protein loading and transformed to a linear value. Linear values were transformed to Log2 value and then median-centered for hierarchical cluster analysis. The Heatmap was generated using correlation distance metric and hierarchical cluster analysis. Protein intensity values are log2 and z-score transformed to remove any technical variation. Proteins changed by FC > 1.2 (Red) FC < 1.2 (Blue) with reference to untreated samples were used for the analysis. The RPPA data used in this analysis can be found at http://www.ncbi.nlm.nih.gov/geo/ (GSE67502). Further Ingenuity Pathway Analysis (IPA) software (http://www.ingenuity.com) was used to analyze cellular signaling pathways for each group of genes under MPS1 inhibition, to predict Top Canonical Pathways and their associated regulators.

### Bioinformatics; survival analysis

Prognostic effect of miR-21, PDCD4, MSH2 expression correlated to patient survival for an independent cohort of 197 GBM patients was analyzed by curating GBM-TCGA database (https://tcga-data.nci.nih.gov). An in-house Glioblastoma Bio Discovery Portal (GBM-BioDP) [33] was used to measure differential expression from three platforms (Affymetrix HGU133A, Agilent G4502A, and HuEx-1_0-st-v2) within the subtypes of GBM (as per *Verhaak et al* [57]) and potential associations with clinical outcome. Multivariate survival analysis was carried out using a Cox Proportional Hazards model taking into joint effect of three covariates - expressions stratified as below and above median, age at diagnosis, and MGMT methylation status. The impact of each covariate was assessed by the covariate's hazard ratio, and its associated *p*-value. A log-rank of < = 0.05 and a *p*-value < = 0.05 were considered statistically significant. This tool also helped us to construct expression correlation heat maps between miRNAs and gene products. GBM-BioDP is a free web-accessible resource that hosts a subset of the glioblastoma TCGA data and enables an intuitive query and interactive display of the resultant data (http://gbm-biodp.nci.nih.gov) [25].

### Real-time PCR-based detection of miR-21 and MPS1-mRNA

Total RNA from GBM cells was extracted using Trizol (Invitrogen), and cDNA was synthesized by using miScript II RT kit (Qiagen, USA) as per manufacturer recommendations. Expression of miR-21-microRNA (Hs04231424_s1) was determined by the TaqMan miRNA-assay (Applied Biosystems, Foster City, CA, USA), and normalized using the 2^^CT-method relative to U6-snRNA (Hs00984809_m1). MPS1-mRNA was quantified by TaqMan-qRT–PCR and normalized to GAPDH (AppliedBiosystems) . All TaqMan-PCRs were performed in triplicates run on Applied Biosystems 7500 Real Time PCR thermal cycler.

### Western blot analysis

Cell pellets were lysed on ice in RIPA buffer (Pierce, Rockford, IL) supplemented with Complete Mini EDTA-free Protease Inhibitor Cocktail (Roche, Indianapolis, IN) and Phosphatase Inhibitor Cocktail (Sigma, St. Louis, MO). (Tumors were lysed in the same buffer using a Homogenizer). Protein concentrations were determined by Bradford assay (Bio-Rad, Hercules, CA). Protein(50ug) was diluted 1:5 in 5X protein loading buffer (Fermentas, Glen Burnie, MD), boiled at 80°C for 5 minutes, electrophoresed on a 4-20% Tris-Glycine gel, and transferred using a Trans-Blot Turbo Transfer System (Bio-Rad, Hercules, CA). Membranes were blocked in 5% Non-fat milk powder (BioRad), incubated with primary antibody overnight at 4°C, incubated with HRP-coupled secondary antibody 1 hour at room temperature, developed with Visualizer Western Blot Detection Kit (Millipore, Billerica, MA), and visualized on a LAS-4000 imager (Fujifilm, Edison, NJ). The following antibodies were used at 1:1000 dilutions: rabbit anti-Smad2/3 (#3102), rabbit-anti- Phospho-Smad2 (Ser465/467)/Smad3 (Ser423/425) (#8828), mouse-anti- MSH2 (#2850), rabbit-anti-PDCD4 (#9535) were from Cell Signaling Technology (CST)., MA, human-anti-MPS1 (05-683, Millipore); mouse anti-actin (MAB 1501R, Millipore). Secondary antibodies, goat anti-rabbit-HRP and goat anti-mouse-HRP (Santa Cruz Biotechnology, Santa Cruz, CA) were used at 1:10,000 dilution. Protein band Intensities were quantitated using Image-J software (NIH).

### Immunofluorescence staining for pSMAD 2/3 and PDCD4

Localization of endogenous pSMAD2/3 and PDCD4 was assessed by immunofluorescent staining. U251 GBM cells were seeded on 4 well chamber slides (Lab-Tek.,Thermo Fischer Scientific) . After attachment and treatment as indicated, cells were fixed in 4% paraformaldehyde (in PBS +0.1% Triton X-100 for permeabilisation) (pH 7.4,) and then blocked with 3% BSA in PBS, followed by overnight incubation with goat polyclonal-anti- pSMAD2/3 (sc-11769, (Santa Cruz Biotechnology, Santa Cruz, CA)) and rabbit-anti-PDCD4 primary antibody (#9535, CST) (1:500 dilution with PBS containing 3% BSA). Alexa flour conjugated AffiniPure donkey anti-goat IgG and goat-anti-rabbit IgG secondary antibodies (Invitrogen) were then applied. Slides were then mounted with fluorescent medium VECTASHIELD containing DAPI (Nuclear stain) (Vector Laboratories Inc., Burlingame, CA) to preserve the fluorescent signal. The localization of endogenous pSMAD2/3 and PDCD4 were visualized and images captured with Olympus FSX100 fluorescent microscope.

### Statistical analysis

Data presented are the mean ± S.D from three independent experiments unless indicated otherwise. All statistical tests were two-sided. For comparisons between groups, a Student's t test was performed and the level of significance was set at P* < 0.05. Analysis was done using MS Excel 2010 software (Microsoft Corp., Washington, USA).

## SUPPLEMENTARY MATERIALS FIGURES


