# Supplementary material for: Modulation of miR-21 signaling by MPS1 in human glioblastoma
Source: Oncotarget. 2015 May 15;7(33):52912–27. doi: 10.18632/oncotarget.4143 (PMC5288158; doi:10.18632/oncotarget.4143)
Supplement: Supplementary file 1 [file oncotarget-07-52912-s001.pdf]

# Modulation of miR-21 signaling by MPS1 in human glioblastoma

**Supplementary Material**

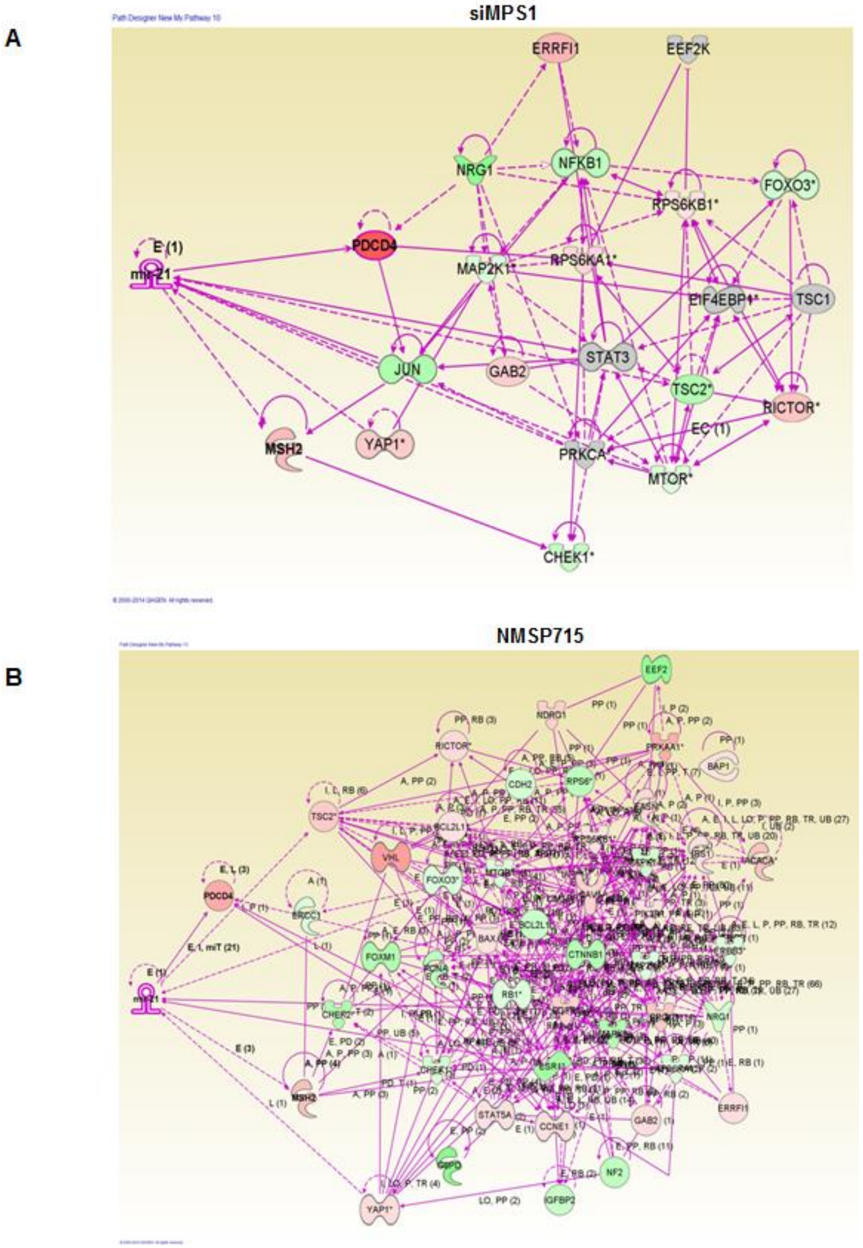


**Supplementary Figure 1: Ingenuity pathway analysis (IPA) predicted molecular interaction network.**

Panel **A, B** represent IPA generated molecular interactions among commonly affected genes (between U251 and U87) with miR-21 in siMPS1 and NMSP715 treated cells

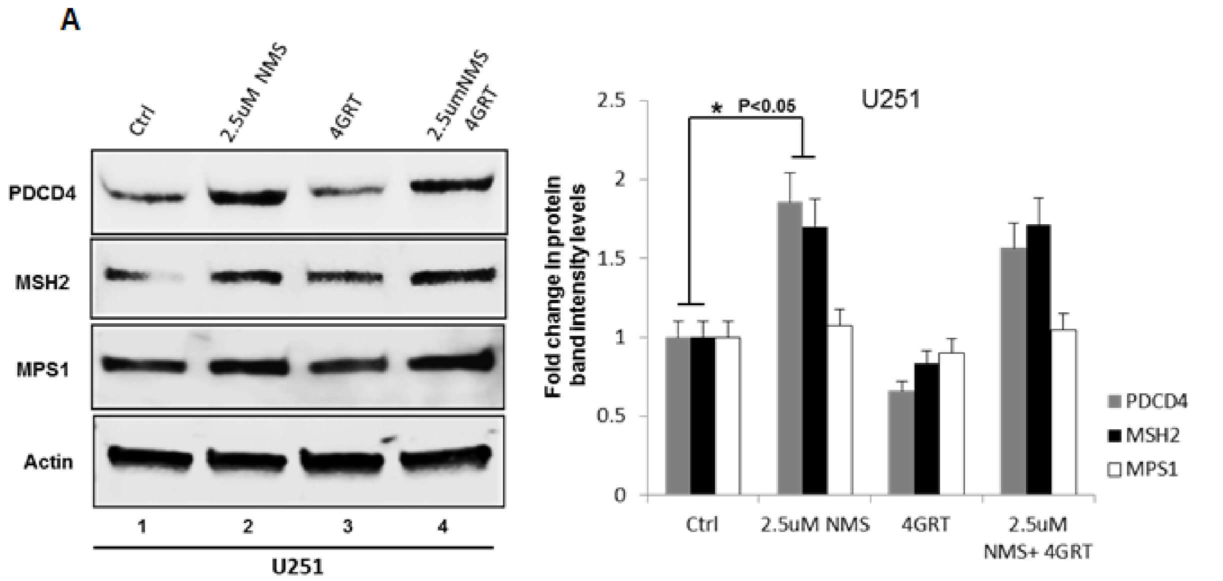


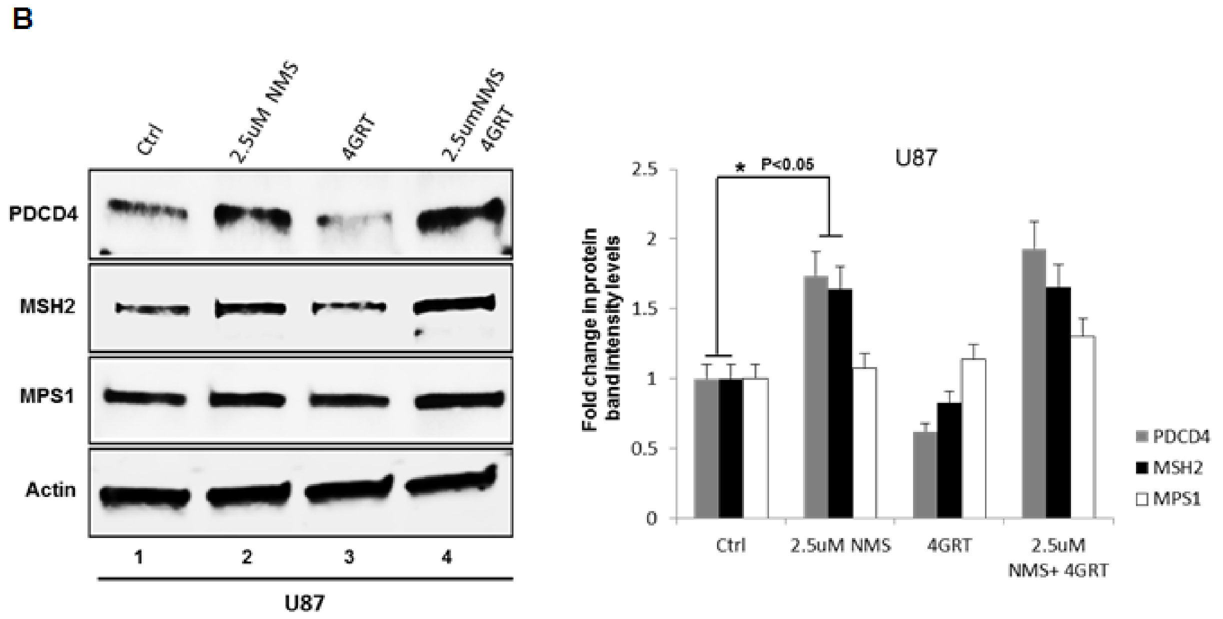


**Supplementary Figure 2: Pharmacologic MPS1 inhibition enhances PDCD4, MSH2 expression *in vitro.***

Panel **(A, B)** represent western blot analysis of PDCD4, MSH2, MPS1 and β-Actin proteins from cell lysates of U251 and U87 treated GBM cells as indicated at 48hr time point, with their corresponding bar graphs of western blots representing the fold change in protein band intensities normalized to β-Actin quantified densitometrically using Image-J software NIH. Data presented are the mean ± S.D. Student's t test was performed and the level of significance * indicate $p<0.05$.

**Predicted MPS1-SMAD3-miR21 pathway:**

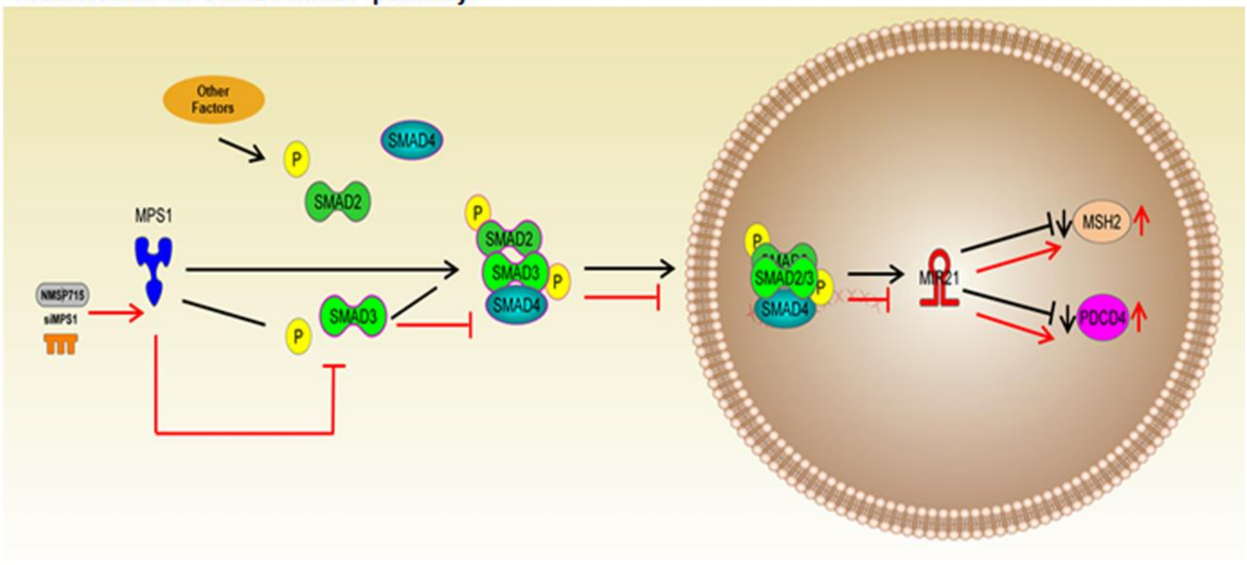


**Supplementary Figure 3: Predicted MPS1 -SMAD3-miR-21 pathway.**

Illustrative image representing MPS1 modulating SMAD3 phosphorylation and its subsequent localization into nucleus as a complex (pSMAD2/3/SMAD4), where it is hypothesized to regulate PDCD4, MSH2 through microRNA- miR-21.

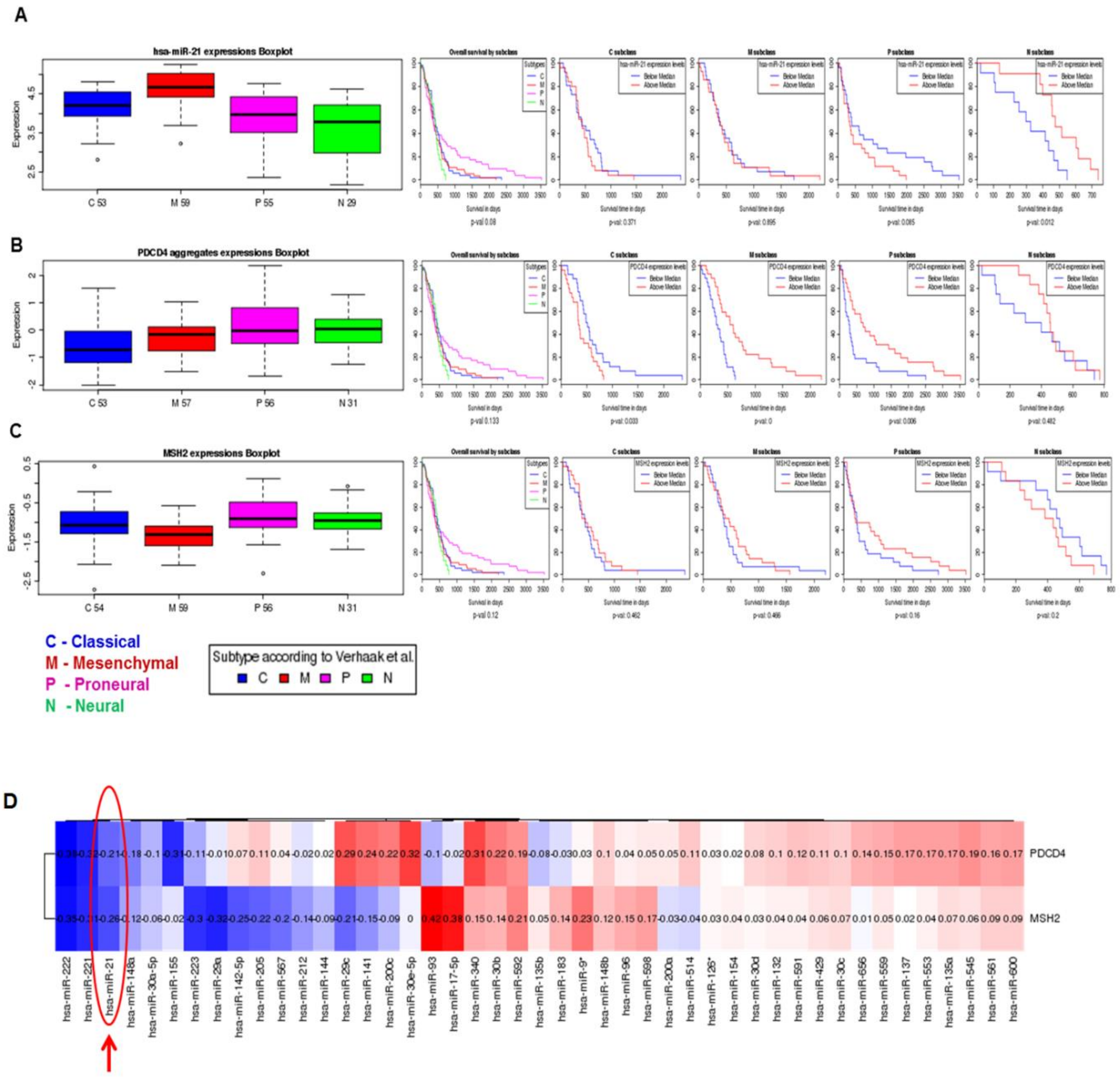


**Supplementary Figure 4: Prognostic effect of miR-21, PDCD4, and MSH2 expression on GBM patient survival.**

Represents BOX plots and univariate Kaplan- Meier survival Curves for below median (blue) and above median (red) gene expression values for miR-21 **(A)** PDCD4 **(B)** and MSH2 **(C)** in different Glioblastoma subtypes: (C)lassical (M)esenchymal , (P)roneural and (N)eural. Glioblastoma Bio Discovery Portal (GBM-BioDP) (http://gbm-biodp.nci.nih.gov) was used to generate this analysis. Panel **(D)** represent expression

correlation heatmaps between miRNAs and gene products (PDCD4, MSH2) among GBM patients (n=197) with the correlation co-efficient (CV) values in the boxes. Red arrow shows MiR21 correlation to PDCD4 (CV: -0.21), MSH2 (CV: -0.26). Color red represents positive correlation, color blue represents negative correlation.
